# Supplementary material for: Evolution and activation mechanism of the flavivirus class II membrane-fusion machinery
Source: Nat Commun. 2022 Jun 28;13:3718. doi: 10.1038/s41467-022-31111-y (PMC9239988; doi:10.1038/s41467-022-31111-y)
Supplement: Supplementary file 1 — Supplementary Information [file 41467_2022_31111_MOESM1_ESM.pdf]

Supplementary Information

Evolution and activation mechanism of the flavivirus class II membrane-fusion machinery

Marie-Christine Vaney[1†], Mariano Dellarole[1,5†], Stephane Duquerroy[1,2†], Iris Medits[3], Georgios Tsouchnikas[3,5], Alexander Rouvinski[1,5], Patrick England[4], Karin Stiasny[3*], Franz X. Heinz[3*] and Felix A. Rey[1*]

[1] Institut Pasteur, Université de Paris, CNRS UMR 3569, Unité de Virologie Structurale, F-[7] 5015 Paris, France.
[2] Université Paris-Saclay, Faculté des Sciences, F-91405 Orsay, France.
[3] Center for Virology, Medical University of Vienna, 1090 Vienna, Austria.
[4] Institut Pasteur, Université de Paris, CNRS UMR 3528, Plateforme de Biophysique Moléculaire, F-75015 Paris, France. ‡
[5]Department of Microbiology and Molecular Genetics, Institute for Medical Research Israel-Canada, The Kuvin Center for the Study of Infectious and Tropical Diseases, The Hebrew University of Jerusalem, Jerusalem, Israel (AR)

†These authors contributed equally

*Correspondence: karin.stiasny@meduniwien.ac.at; Franz.X.Heinz@meduniwien.ac.at; felix.rey@pasteur.fr (F.A.R)

Supplementary Figure 1

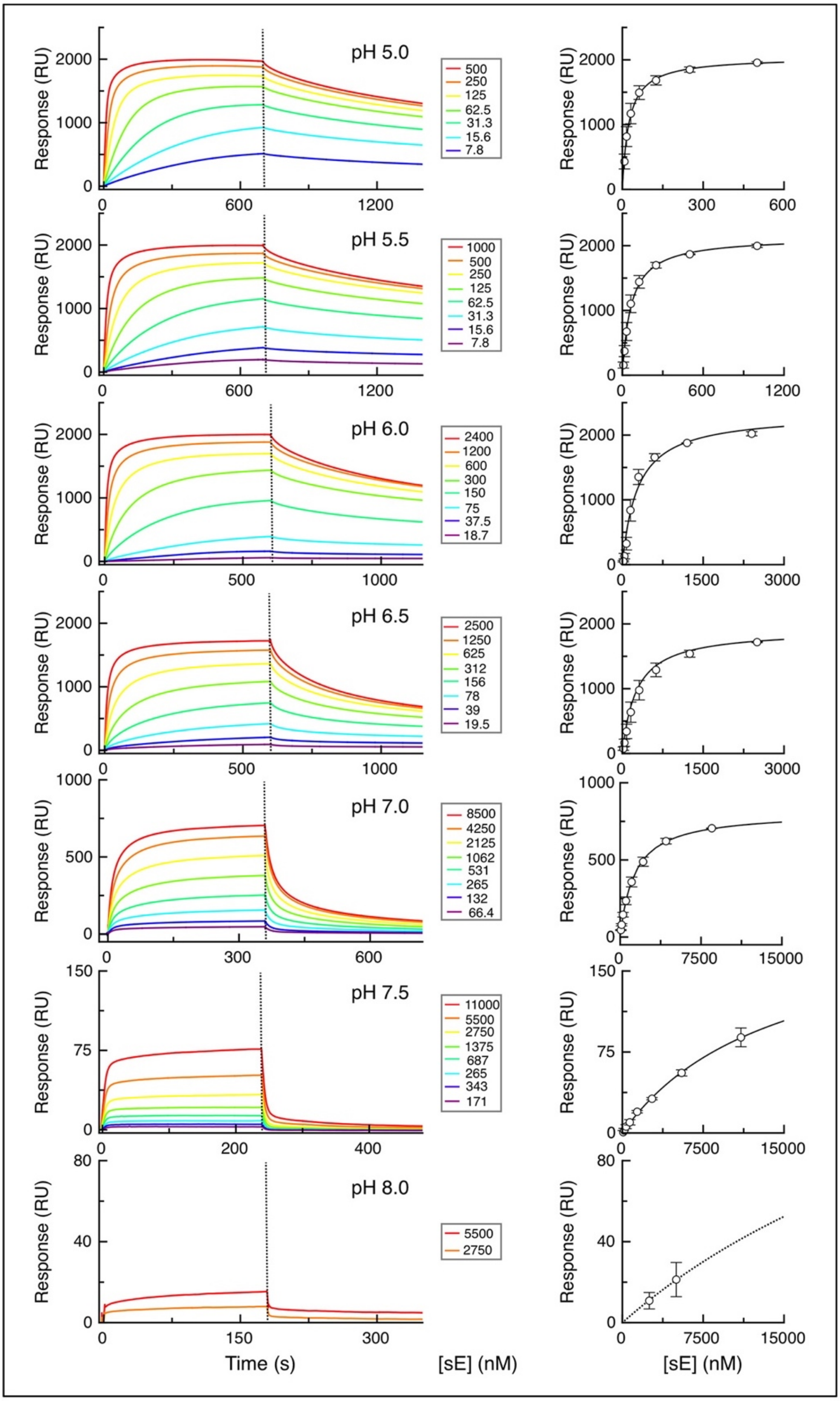

Supplementary Fig. 1. Surface plasmon resonance titration experiments at different pHs.

Left column: pr/sE association and dissociation kinetics corresponding to injections of sE on immobilized pr and equilibrated at pH 5.0, 5.5, 6.0, 6.5, 7.0, 7.5 and 8.0. The dotted line shows the switch from association to dissociation kinetics. Right column: plots of the maximum SPR association intensity (Rmax) recorded for each pH as a function of sE concentration. The curve is the fit of the data to the equilibrium single-site binding model provided by the SPR software. The measurements were reproduced twice. Error bars display the mean ± SD for each individual data point. For pH 8.0, the dissociation constant was estimated by using the saturation Rmax data from pH 7.5 measurement. Source data are provided as a Source Data file.

Supplementary Figure 2

Panel A: pr-alignment

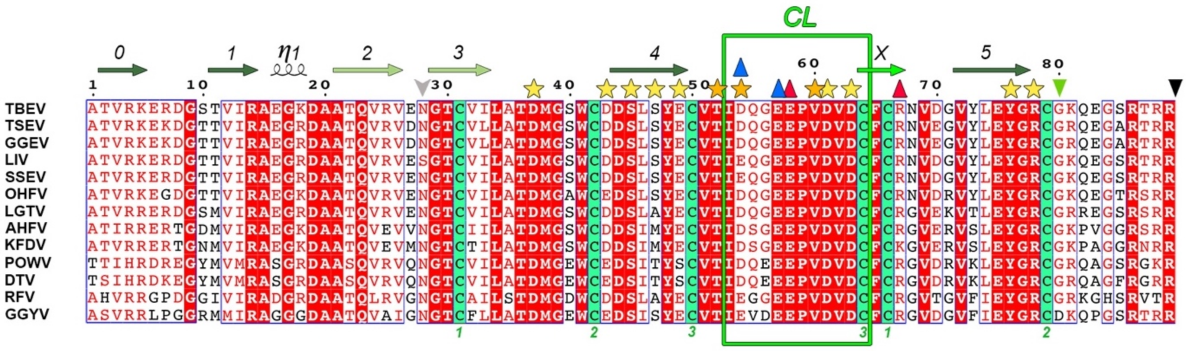

intra-protomer interactions with E domain II
intra-protomer interactions with E fusion loop
inter-protomer interaction with domain I
inter-protomer interaction with domain III

last residue seen in density in TBEV pr/sE structure
furin cleavage site
glycans

Supplementary Figure 2

Panel B: E-alignment

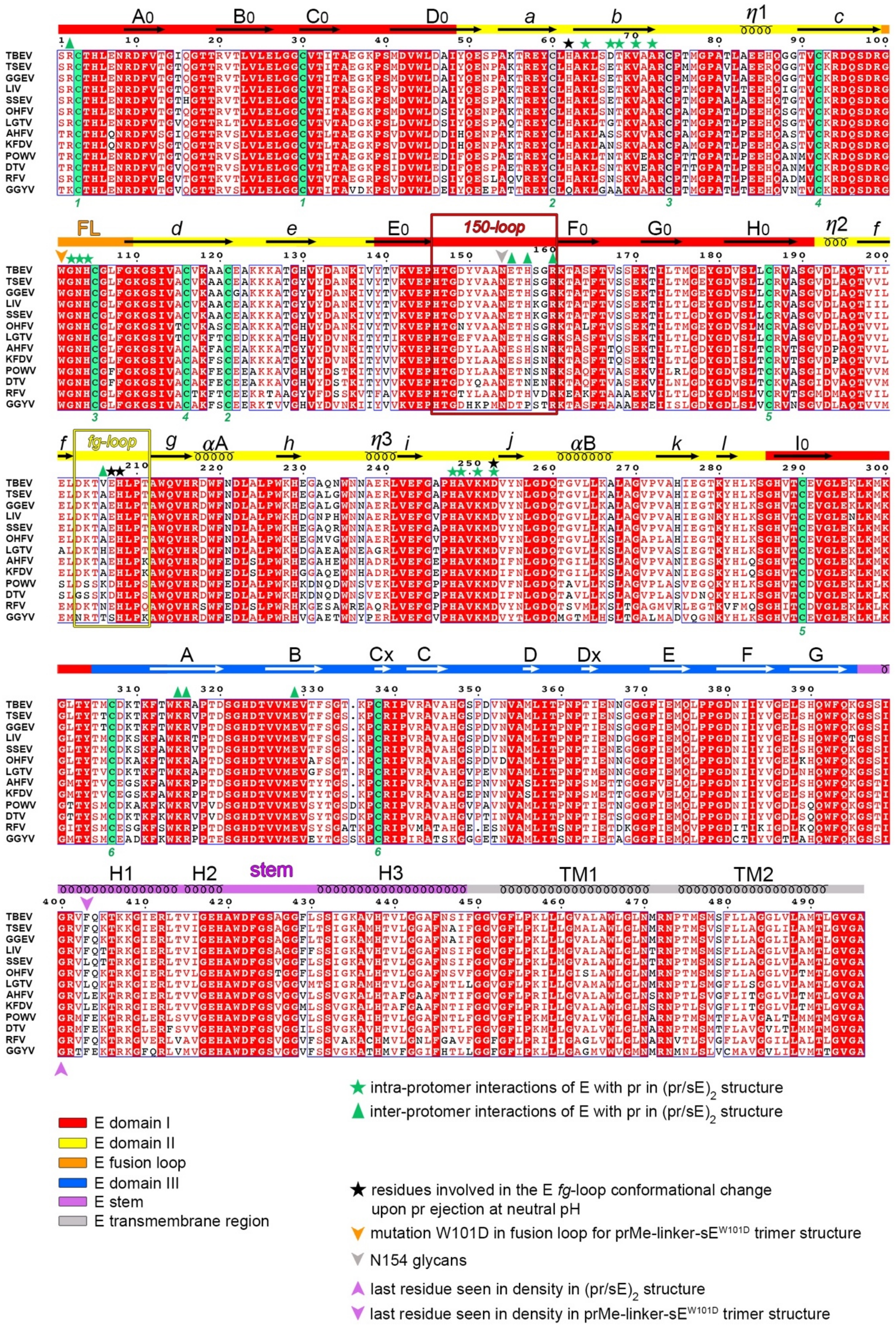

Supplementary Fig. 2. pr-E interacting residues mapped onto a multiple amino acid sequence alignment from tick-borne viruses infecting vertebrates.

Panel A: pr alignment. Panel B: E alignment. The interacting residues are marked by symbols coded to indicate where they interact in the opposite protein, as indicated in the key below the alignment. The secondary structure elements of TBEV pr and E are colored above the sequences together with the structural elements. A red background highlights identical residue. The disulfide bonds are conserved among all the flaviviruses (green background) and are numbered in green below the sequences. The glycans (N*X*T or N*X*S) are indicated with a grey arrowhead above the sequence alignment.

Supplementary Figure 3

Panel A: pr-alignment

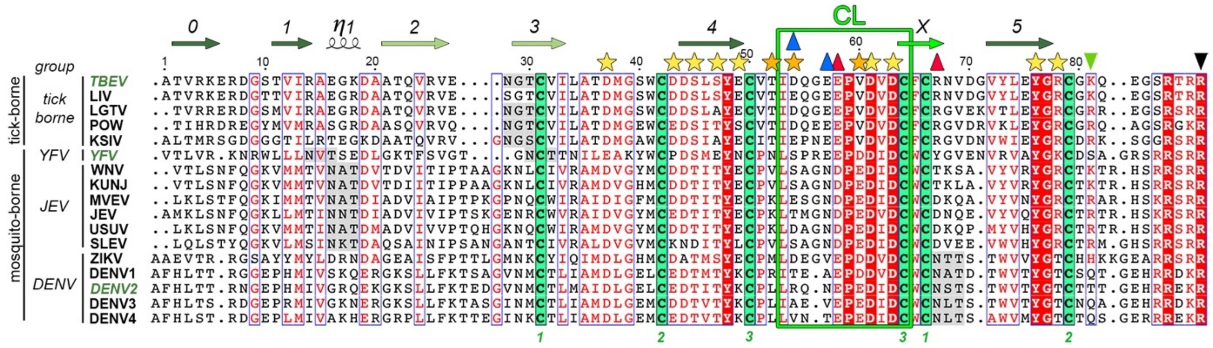

intra-protomer interactions with E domain II

intra-protomer interactions with E fusion loop

inter-protomer interactions with E domain I

inter-protomer interactions with E domain III

last residue seen in density in $(pr/sE)_2$ structure

furin cleavage site

Panel B: E-alignment

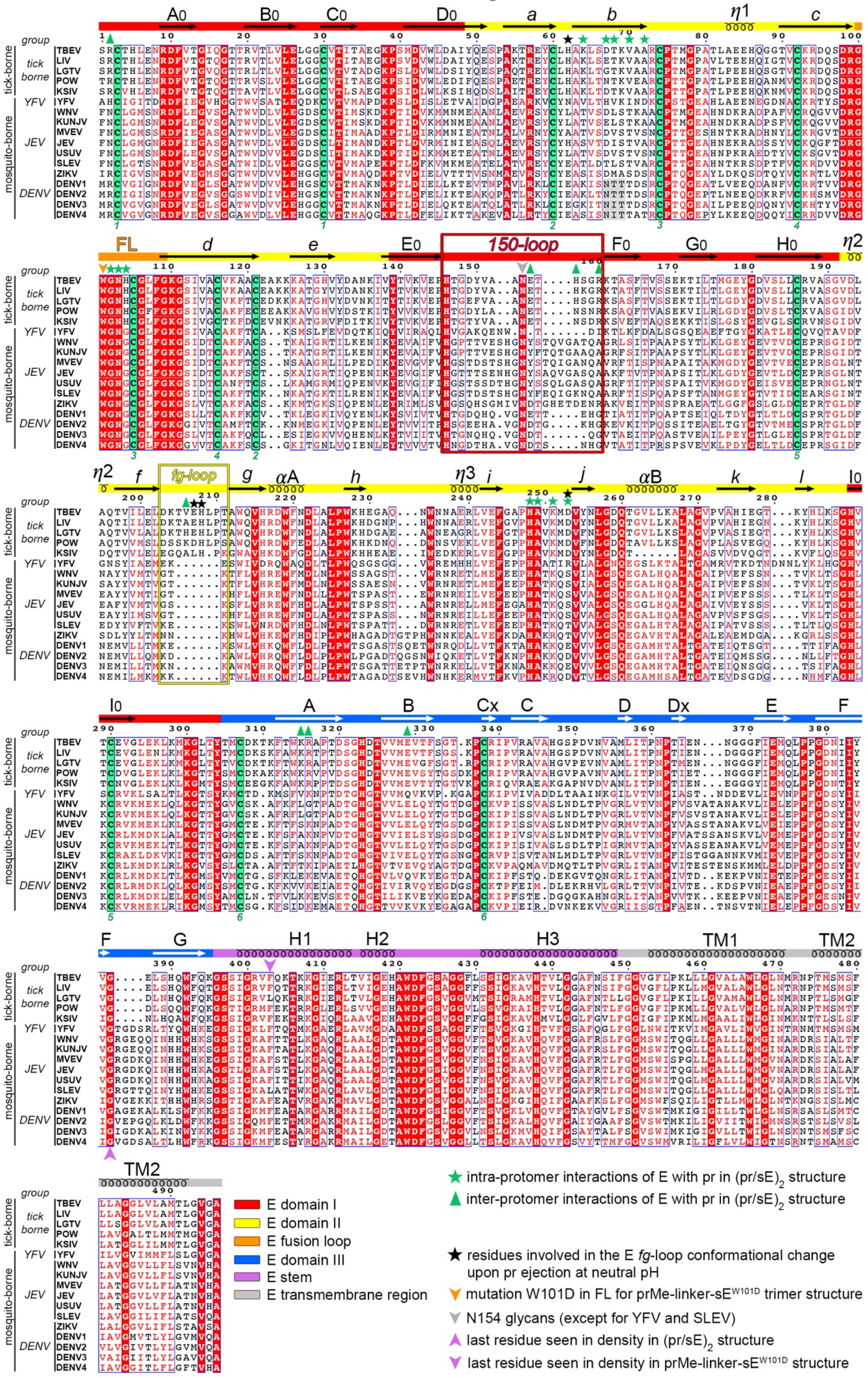

Supplementary Fig. 3. pr-E interacting residues mapped onto a multiple sequence alignment across human-pathogenic flaviviruses.

Panel A: pr alignment. Panel B: E alignment. The symbols used in the annotations of Supplementary Fig. 2 are also used here. The glycans (N*X*T or N*X*S) are indicated with a grey background for the pr sequence alignment and with a grey arrowhead above the E sequence alignment. The analyzed flaviviruses are: TBEV, LIV, LGTV, POWV, KSIV (tick-borne group); YFV for the YFV group; WNV, KUNJ, MVEV, JEV, USUV and SLEV for the JEV group; ZIKV and DENV serotypes 1–4 for the DENV group.

Supplementary Figure 4

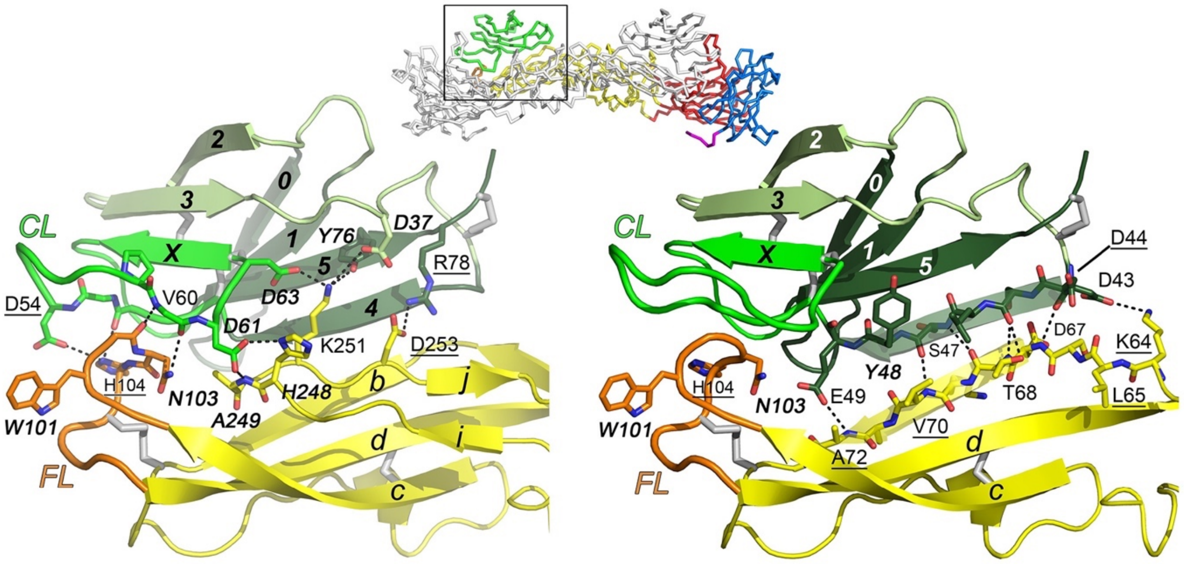

Supplementary Fig. 4. Intra-protomer pr/E interactions.

The $(pr/sE)_2$ dimer is represented in the inset as in Fig. 3a, but rotated by 180 degrees about the molecular 2-fold axis, with a box indicating the closeups shown in the two panels below. The left panel indicates the polar/electrostatic bonds between sidechains of the E fusion loop and *ij* hairpin with pr, while the right panel highlights the main chain β-interactions between E domain II strand *b* and pr β_4. Residues conserved in tick-borne flaviviruses are underlined, while those conserved across all flaviviruses are in bold and italic.

Supplementary Figure 5

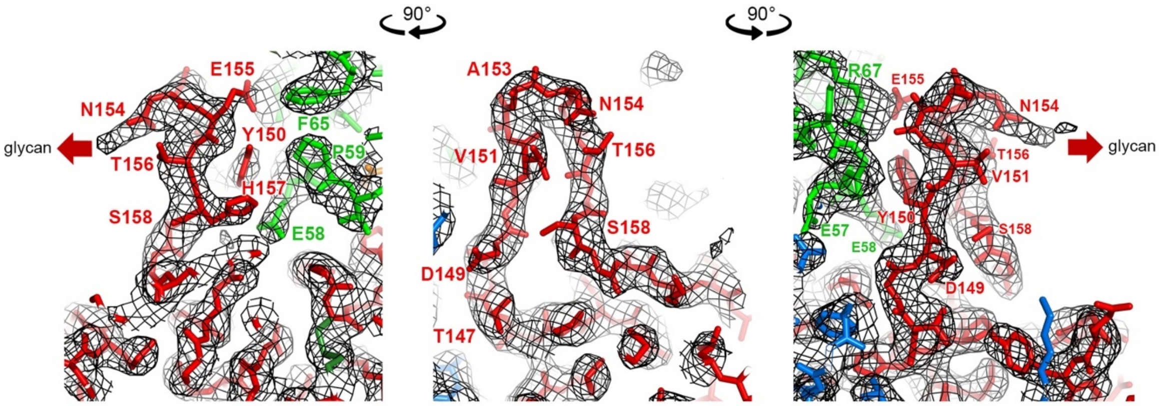

Supplementary Fig. 5. Continuous, traceable experimental electron density for the 150-loop in the crystals.

The 150-loop is viewed from different angles as indicated, showing the electron density from the simulated annealing 2Fo-Fc composite map contoured at 1 sigma level. Despite displaying clear, continuous electron density for the whole loop and for the Asn154 side chain, the crystal shows no density for the attached glycan, indicating that it is highly mobile, contrary to the structure in the absence of pr (PDB 1SVB). The red arrow on the right panel indicates the potential orientations of the missing Asn154 glycan, away from the interaction with pr.

Supplementary Figure 6

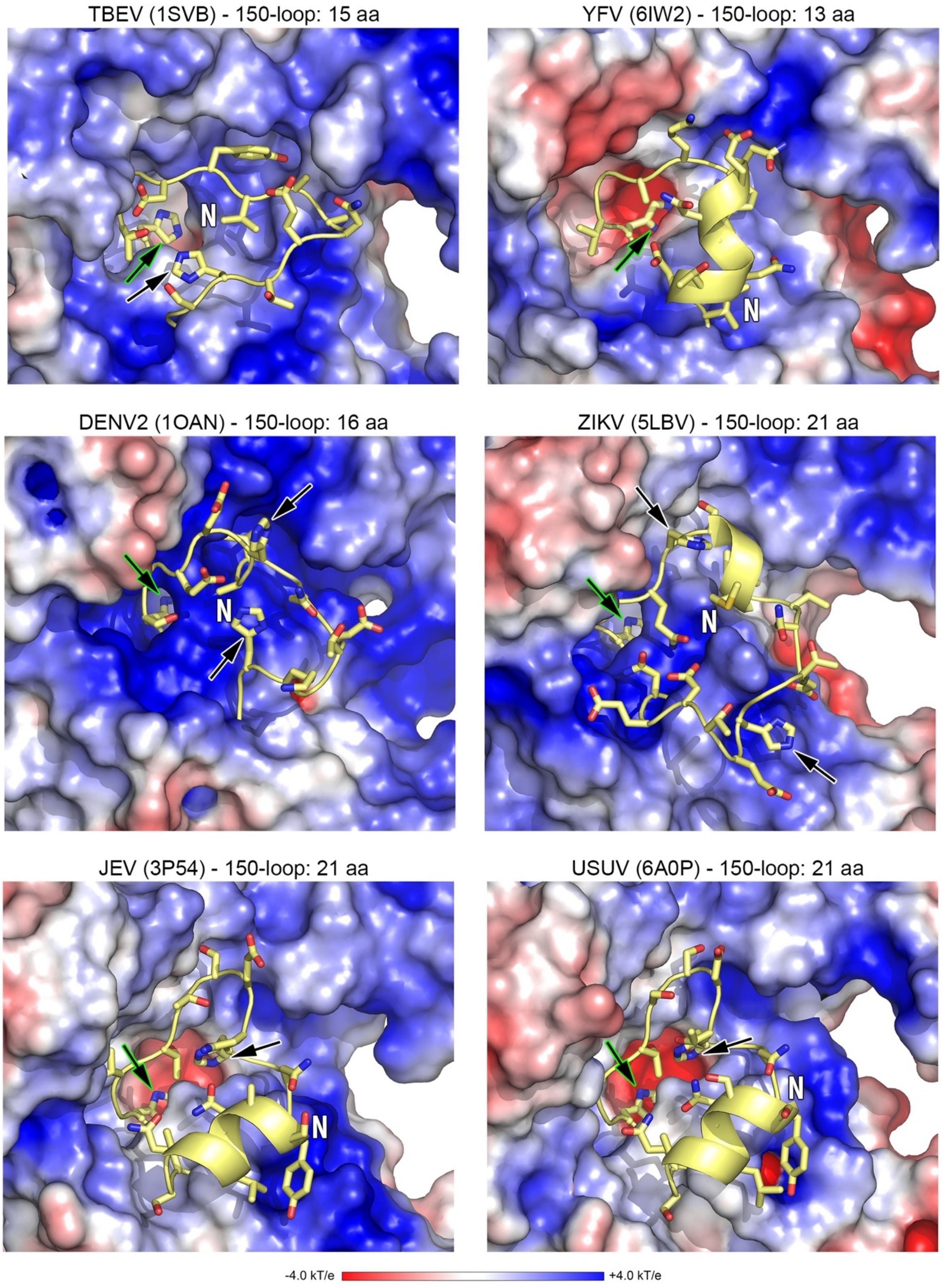

Supplementary Fig. 6. Closeups of the 150-loop packing against the N-ter region in the E proteins of selected flaviviruses.

This Figure zooms into the left-side of the sE dimers represented at pH 7.5 in Fig. 5, right column. The 150-loop was removed to calculate the electrostatic potential at the surface in contact with the lid in mature particles. The model of the 150-loop is superposed, as ribbon for its main chain and sticks for its side chains, to illustrate the interactions made in each virus. The location of the N-terminus on this surface is labeled with “N”. Black arrows point to histidine residues. A green outline on the arrow highlights a strictly conserved histidine (His146 in TBEV, see Supplementary Fig. 3) located at the base of the 150-loop, at the base of the “hinged-lid” discussed in the text. The number of amino acids forming the 150-loop is indicated for each virus. Despite the variability in amino acid sequence, all the structures show the 150-loop interacting with the charged N-terminus of the E protein.

Supplementary Figure 7

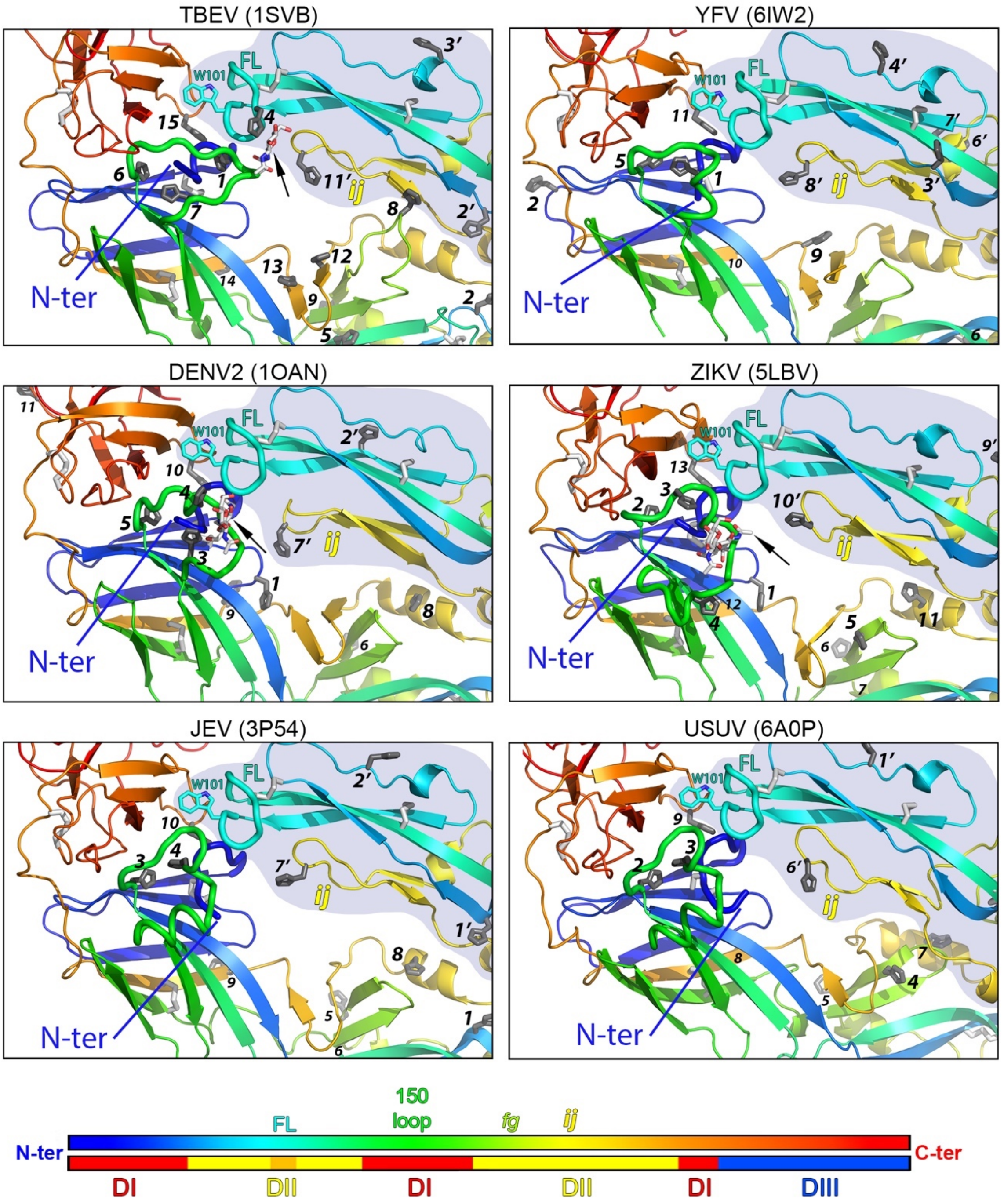

Supplementary Fig. 7. Histidine residues cluster at the 150-loop / N-ter region interface in the E proteins of various flaviviruses.

Each panel shows a ribbon representation of the sE dimer for each of the flaviviruses displayed in Fig. 5 and in the same orientation, centered on the sE dimer interface. The sE subunit on the top-right of each panel is shown on a grey background to outline the dimer interface, with the fusion loop (cyan, labeled FL) and Trp101 drawn as sticks and labeled. The *ij* hairpin (yellow) is also labeled. The N-terminus (dark blue) is labeled with a blue bar pointing to it in the bottom-

left subunit. The sE ribbon is ramp-colored from N-ter (dark blue) to C-ter (red) through cyan, green, yellow and orange. This color scheme is indicated in the color bar underneath, displayed together with a second colored bar showing the correspondence with the domain colors defined in Fig. 2a. In this scheme, the 150-loop is green (labeled over the color code bar), shown as a green tube on top of the blue N-terminus in the ribbon diagrams of the sE dimer. The attached glycan, when present, is displayed as sticks color-coded as atom type (white, red and blue for carbon, oxygen and nitrogen, respectively), marked with a thin black arrow. Represented as sticks are also the disulfide bonds (in light grey), and the histidine residues (dark grey). In order to further highlight them, the histidine residues were numbered in sequence according to their location in the primary structure (primed numbers were used in the top-right subunit). Note that despite the overall absence of amino acid sequence conservation across flaviviruses, as shown in Supplementary Fig. 3, there is clustering of histidine residues at the 150-loop and its surroundings.

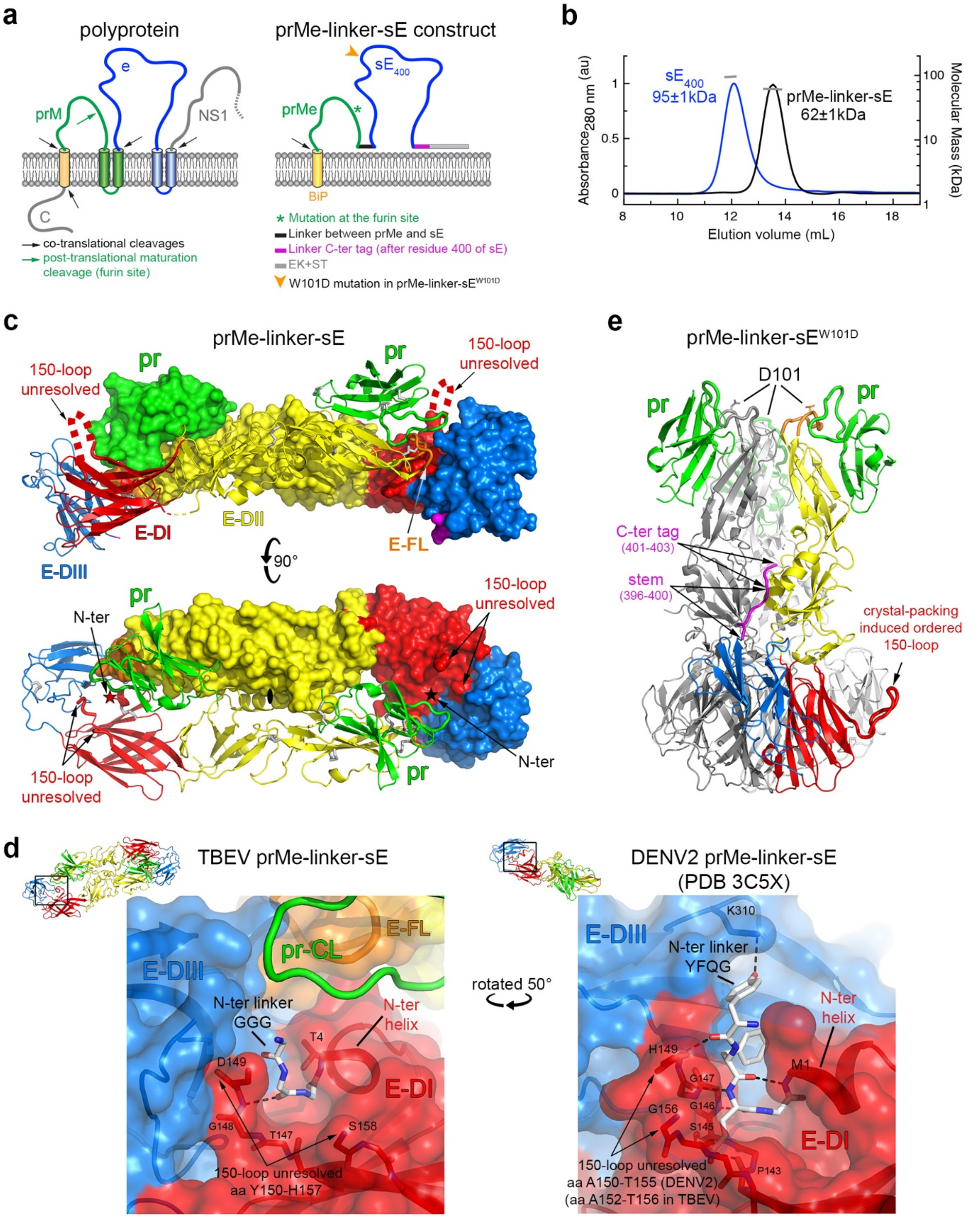

Supplementary Fig. 8. X-ray structures of the prMe-linker-sE and prMe-linker-sE^{W101D} constructs.

(a) Schematic representation of the N-terminal, "structural protein" region of the flavivirus precursor polyprotein (left panel) and the truncations and linker insertion made to obtain a single-chain soluble prMe-linker-sE construct (right panel). The linker replaces the two C-

terminal trans-membrane segments of prM and connects directly to the E N-terminus. In addition, the furin cleavage site (green arrow, left panel) was abrogated by a mutation (green star in the right panel) (see Methods). The orange arrowhead in the right panel indicates the extra mutation in the fusion loop introduced to make the prMe-linker-sE^{W101D} construct. The C-terminal tag after residue E 400 is colored pink and grey as indicated in the inset (EK – enterokinase cleavage site; ST – double strep-tag). **(b)** SEC-MALS profiles at pH 8 of the single-chain construct compared to that of wild- type sE, showing that prMe-linker-sE behaves as a monomer, contrary to sE, which is dimeric at this pH. Note that the presence of pr in the prMe-linker-sE monomer makes this construct elute at the expected volume (~ 13.8 ml) and does not display the aberrant behavior of the sE monomer, which elutes ~ 17.5 ml in the same column (Fig. 1a, middle panel). **(c)** Two orthogonal views of the prMe-linker-sE structure, represented as in Fig. 2a, with the protomers colored according to domains, one displayed as surface and the other as ribbon. The main difference is that the 150-loop is disordered (indicated by dashed red ribbons in the top panel, and by arrows pointing to the site where the electron density in the crystals is lost in the bottom panel), and the presence of part of the linker extending from the sE N-terminus (marked with a red star in the bottom panel). **(d)** Closeups showing the N-terminal region of sE in TBEV prMe-linker-sE dimer structure (PDB 7QRF, this work) (left panel) and in DENV2 prMe-linker-sE monomer structure reported previously (PDB 3C5X) (right panel). The E subunit is displayed in transparent surface with the N-terminal linker residues as sticks colored by atom-type. For TBEV, 3 residues (GGG) of the 14-aa linker protrude out of the surface while for DENV2, four residues (YFQG) from the 8-aa linker prevent ordering of the 150-loop. In both crystals, the density break is between the two hinges of the lid (black arrows) and in both cases the visible amino acids of the linker make a network of hydrogen bonds with residues of the N-terminal hinge of the 150-loop. **(e)** Structure of prMe-linker-sE^{W101D} (PDB 7QRG, this work). The trimer is shown with one sE subunit colored as in (c) and the others in in light and dark grey, except for pr in green in all three protomers. The site of the W101D mutation in the fusion loop is indicated. The stem (residues 396-400) and the visible part of the tag (residues 401-403 in this construct) are in magenta.

Supplementary Table 1. E-pr binding parameters.

pH	K_D (nM)	$\Delta G_{binding}$ (kcal/mol)
5.0	25.15 ± 1.12	-10.18 ± 0.03
5.5	66.05 ± 3.42	-9.6 ± 0.03
6.0	286.6 ± 40.6	-8.8 ± 0.08
6.5	348.75 ± 20.6	-8.7 ± 0.03
7.0	1396.5 ± 73.0	-7.8 ± 0.03
7.5	10634 ± 841.4	-6.7 ± 0.05
8.0	≧ 34000	≧ -6.0

G is calculated as RTln(K_D). Errors are given from at least two experiments performed in 50 mM Tris-HCl, 50 mM MES (pH 5.0, 5.5, 6.0, 6.5, 7.0, 7.5, 8.0) with 150 mM NaCl. Source data are provided as a Source Data file.

Supplementary Table 2. Crystallographic and refinement statistics

Structures	pr/sE	prMe-linker-sE dimer	prMe-linker-sE^{W101D} trimer
**PDB code**	**7QRE**	**7QRF**	**7QRG**
**Crystallisation conditions**			
Protein concentration (mg/mL)	pr 0.7 / sE 1.2<br>mix molar ratio 2:1	1.5	0.9
Crystallization conditions	0.2M $(NH4)_2SO_4$<br>0.1M Na acetate pH 4.6<br>28% (v/v) PEG MME 550<br>2.8% (v/v) tert-Butanol	0.2M $Li(SO_4)$<br>0.1M Na citrate pH 3.5<br>28% (v/v) PEG 400	0.1M Na Malonate<br>0.1M HEPES pH 6.7<br>18.9% (w/v) PEG 3350
Crystallization pH	4.6	3.5	4.5<br>(measured in drop)
Cryo-protectant	None	16% ethylene glycol in 84%<br>of crystallization solution	22% glycerol in 78%<br>of crystallization solution
**Data Collection**$^{\$}$			
Synchrotron/Beamline	SOLEIL PX1	ESRF ID23-1	ESRF ID29
Detector	Pilatus 6M	Pilatus 6M-F	Pilatus 6M
Space group	P 4_3 3 2	H 3 2	P 3 2 1
Unit cell a, b, c (Å)	164.4, 164.4, 164.4	155.6, 155.6, 161.6	97.9, 97.9, 115.2
α, β, γ (°)	90, 90, 90	90, 90, 120	90, 90, 120
**Resolution (Å)**	**2.7** (2.79-2.7)	**2.28*** (2.43-2.28)	**2.8** (2.95-2.8)
Rmerge (%)	24.2 (474)	9.6 (262)	23.8 (273)
Rmeas (%)	24.9 (490)	9.9 (270)	28.6 (325)
Rpim (%)	5.9 (122)	2.3 (63)	11.7 (131)
<I/σ(I)>	13.8 (0.9)	19.8 (1.3)	6.3 (0.7)
$CC_{1/2}$	99.8 (33.2)	99.8 (32.3)	98.8 (30.6)
Completeness (spherical) (%)	100 (100)	84.3 (24.1)	98.9 (99.4)
Completeness (ellipsoidal) (%)*		95.0 (64.8)	
Redundancy	32.9 (30)	19.5 (18)	5.7 (5.9)
**Structure Determination**			
Oligomeric state in crystal	dimer	dimer	trimer
N° of molecules/au	1 pr + 1 sE	1 prM*ecto*-sE	1 prM*ecto*-sE*
**Refinement**			
Resolution (Å)	49.54-2.7	28.42-2.28$^{£}$	49.2-2.8
N° of reflections (Work/Free)	21399 / 1071	28965 / 1487	15902 / 826
Rwork (%) / Rfree (%)	18.8 / 24.9	18.1 / 21.3	22.8 / 26.4
<B> atomic factors $(Å)^2$	85.1	80.8	79.9
Use of TLS	yes	yes	yes
N° of atoms			
Protein	3658	3564	3642
Heterogen	53	53	12
Water	22	74	14
R.m.s. deviations			
Bond lengths (Å)	0.01	0.01	0.007
Bond angles (°)	1.26	1.16	0.93
Ramachandran statistics$^{\|\|}$			
Favoured (%)	94.12	97.35	93.63
Allowed (%)	5.67	2.65	6.37
Outliers (%)	0.21	0	0

PDB, Protein Data Bank; PEG, polyethylene glycol; au, asymmetric unit

$ Highest-resolution shell is shown in parenthesis

Rmerge = $\Sigma_h \Sigma_i (|\langle I_h \rangle - I_{h,i}|) / \Sigma_h \Sigma_i I_{h,i}$ (where h are the unique reflections and i their symmetry-equivalent)

Rwork = Σ|Fo − Fc|/Σ|Fo|; Rfree = Σ|Fo − Fc|/Σ|Fo| using 5% of the Fo selected randomly

<I/σ(I)>, mean empirical signal-to-noise ratio; CC(1/2), mean half-set correlation coefficient; r.m.s., root mean square

* Data collection statistics are from Staraniso; £ Structure refined against corrected intensities from anisotropy

TLS, parameterization describing translation, libration and screw-rotation to model anisotropic displacements

|| Ramachandran statistics were calculated with MolProbity

Supplementary Table 3.
Polar and ionic interactions between pr and E in TBEV complexes.

	sE domains		pr/sE DIMER			prMe-linker-sE DIMER			prMe-linker-sE^{W101D} TRIMER			pr secondary structure
			sE	dist (Å)	pr	sE	dist (Å)	pr	sE	dist (Å)	pr	
Subunit E: intra-protomer interactions	domain II	*b*-strand	**LYS 64 [NZ]**	**3.2**	**ASP 43 [OD2]**				**LYS 64 [NZ]**	**3.5**	**ASP 43 [OD2]**	β3-β4 loop
			ASP 67 [N]	*3.1*	*ASP 43 [O]*	*ASP 67 [N]*	*3.0*	*ASP 43 [O]*	*ASP 67 [N]*	*3.1*	*ASP 43 [O]*	β3-β4 loop
			ASP 67 [OD1]	3.6*	SER 45 [OG]	ASP 67 [OD1]	3.9*	ASP 43 [OD1]	*ASP 67 [OD1]*	*3.5*	*ASP 43 [O]*	β3-β4 loop
						ASP 67 [N]	*3.8**	*SER 45 [OG]*				
			THR 68 [N]	*3.3*	*SER 45 [O]*	*THR 68 [N]*	*3.2*	*SER 45 [O]*	*THR 68 [N]*	*3.0*	*SER 45 [O]*	β4
			THR 68 [OG1]	*3.1*	*SER 45 [O]*	*THR 68 [OG1]*	*3.3*	*SER 45 [O]*	*THR 68 [OG1]*	*3.1*	*SER 45 [O]*	β4
			THR 68 [O]	*2.8*	*SER 47 [N]*	*THR 68 [O]*	*3.0*	*SER 47 [N]*	*THR 68 [O]*	*2.9*	*SER 47 [N]*	β4
									THR 68 [OG1]	3.5	ARG 78 [NH2]	β4
			VAL 70 [N]	*2.9*	*SER 47 [O]*	*VAL 70 [N]*	*2.9*	*SER 47 [O]*	*VAL 70 [N]*	*2.9*	*SER 47 [O]*	β4
			ALA 72 [N]	*3.1*	*GLU 49 [OE1]*							β4
		FL	*GLY 102 [O]*	*2.8*	*VAL 60 [N]*	*GLY 102 [O]*	*3.1*	*VAL 60 [N]*	*GLY 102 [O]*	*3.2*	*VAL 60 [N]*	
			ASN 103 [ND2]	*3.3*	*VAL 60 [O]*	*ASN 103 [ND2]*	*3.3*	*VAL 60 [O]*	*ASN 103 [ND2]*	*3.4*	*VAL 60 [O]*	
			**HIS 104 [ND1]**	**3.4**	**ASP 54 [OD1]**				**HIS 104 [NE2]**	**3.6**	**ASP 54 [OD1]**	*capping loop*
									**HIS 104 [NE2]**	**3.7**	**ASP 54 [OD2]**	
			HIS 104 [ND1]	*2.8*	*THR 52 [O]*	*HIS 104 [ND1]*	*2.9*	*THR 52 [O]*				
						HIS 104 [ND1]	*3.9**	*THR 52 [N]*				
		ij-loop	**HIS 248 [ND1]**	**2.8**	**ASP 61 [OD1]**	**HIS 248 [ND1]**	**2.8**	**ASP 61 [OD1]**	**HIS 248 [ND1]**	**2.8**	**ASP 61 [OD1]**	*capping loop*
						**HIS 248 [ND1]**	**4.0**	**ASP 61 [OD2]**	**HIS 248 [ND1]**	**3.5**	**ASP 61 [OD2]**	*capping loop*
			ALA 249 [N]	*3.7**	*ASP 61 [O]*	*ALA 249 [N]*	*3.7**	*ASP 61 [O]*	*ALA 249 [N]*	*3.6**	*ASP 61 [O]*	*capping loop*
			ALA 249 [N]	*3.2*	*ASP 61 [OD2]*	*ALA 249 [N]*	*2.8*	*ASP 61 [OD2]*	*ALA 249 [N]*	*3.2*	*ASP 61 [OD2]*	*capping loop*
			**LYS 251 [NZ]**	**3.9**	**ASP 37 [OD1]**	**LYS 251 [NZ]**	**3.6**	**ASP 37 [OD1]**	**LYS 251 [NZ]**	**3.6**	**ASP 37 [OD1]**	β3-β4 loop
									**LYS 251 [NZ]**	**3.9**	**ASP 37 [OD2]**	β3-β4 loop
			**LYS 251 [NZ]**	**2.8**	**ASP 63 [OD2]**	**LYS 251 [NZ]**	**2.8**	**ASP 63 [OD2]**				*capping loop*
												capping loop
			LYS 251 [NZ]	3.3	TYR 76 [OH]	LYS 251 [NZ]	3.0	TYR 76 [OH]				β5
			**ASP 253 [OD1]**	**3.1**	**ARG 78 [NH1]**	**ASP 253 [OD1]**	**3.0**	**ARG 78 [NH1]**	**ASP 253 [OD1]**	**2.7**	**ARG 78 [NH1]**	β5
			**ASP 253 [OD2]**	**3.9**	**ARG 78 [NH1]**	**ASP 253 [OD2]**	**3.7**	**ARG 78 [NH1]**				β5
									VAL 254 [O]	*3.5*	*ARG 78 [NH1]*	β5
Subunit E: inter-protomer interactions	domain I	N-ter	*ARG 2 [N]*	*3.5*	*GLU 58 [OE1]*	no interaction			NA			*capping loop*
		150-loop (E0-F0 loop)	**GLU 155 [OE1]**	**2.9**	**ARG 67 [NE]**	E 150-loop disordered			NA			βX
			**GLU 155 [OE2]**	**3.4**	**ARG 67 [NE]**							βX
			**GLU 155 [OE2]**	**3.0**	**ARG 67 [NH2]**							βX
			**HIS 157 [ND1]**	**3.6**	**GLU 58 [OE1]**							*capping loop*
			**HIS 157 [ND1]**	**3.2**	**GLU 58 [OE2]**							*capping loop*
			**ARG 160 [NH2]**	**3.4**	**GLU 58 [OE1]**	**ARG 160 [NH2]**	**3.5**	**GLU 58 [OE1]**				*capping loop*
						**ARG 160 [NH2]**	**3.1**	**GLU 58 [OE2]**				*capping loop*
	dom II	*fg*-loop	*VAL 206 [O]*	*3.6**	*ASP 44 [OD2]*	*VAL 206 [O]*	*3.6**	*ASP 44 [OD2]*	NA			β4
	domain III	A-strand	*LYS 315 [NZ]*	*3.7**	*GLN 55 [O]*	*LYS 315 [NZ]*	*3.0*	*GLN 55 [O]*	NA			*capping loop*
			LYS 315 [NZ]	*3.9**	*GLY 56 [O]*							
			**ARG 316 [NH2]**	**3.2**	**ASP 54 [OD1]**	**ARG 316 [NH2]**	**3.2**	**ASP 54 [OD2]**				
			**ARG 316 [NH1]**	**3.4**	**GLU 57 [OE2]**	**ARG 316 [NH2]**	**3.5**	**GLU 57 [OE2]**				
						**ARG 316 [NE]**	**3.4**	**GLU 57 [OE2]**				
			GLU 329 [OE1]	***3.1***	***GLU 57 [OE2]***	***GLU 329 [OE1]***	***3.0***	***GLU 57 [OE2]***				
						GLU 329 [OE2]	***3.4***	***GLU 57 [OE1]***				
			GLU 329 [OE2]	***3.0***	***GLU 57 [OE2]***	***GLU 329 [OE2]***	***3.0***	***GLU 57 [OE2]***				

Polar contacts computed with PISA.
Distances cut-off: Hydrogen bonds distances, 3.5Å; Salt bridges distances, 4Å; * H-bonds weaker, between 3.5Å and 4.1Å.
In italics: main chain atoms involved in H-bonds; **In bold**: salt bridges; ***In italics and bold***: acidic interactions.
NA: non applicable.

Supplementary Table 4. Differences in interactions between E protomers in $(pr/sE)_2$ and sE dimer structures.

pr/sE dimer (PDB 7QRE)			sE dimer (PDB 1SVB)		
**N-ter**			**N-ter**		
sE-prot2 Arg2 (mc)	H-bond	pr Glu58	sE-prot2 Arg2 (mc)	H-bond	sE-prot2 Glu144
sE-prot2 His5	H-bond	sE-prot1 Gly102 (mc)	sE-prot2 His5	H-bond	sE-prot2 Val151 (mc)
sE-prot1 His62	H-bond	sE-prot2 Glu 207	sE-prot1 His62	No H-bond	
**FL**			**FL**		
sE-prot1 Gly102 (mc)	H-bond	pr Val60 (mc)	sE-prot1 Gly102 (mc)	H-bond	sE-prot2 Ala153 (mc)
sE-prot1 Gly102 (mc)	H-bond	pr Val60 (mc)			
sE-prot1 His104	salt-bridge	pr Glu54	sE-prot1 His104	H-bond	sE-prot2 Glu155
**150-loop**			**150-loop**		
sE-prot2 Glu144	salt-bridge	sE-prot2 Arg160	sE-prot2 Glu144	salt-bridge	sE-prot2 Arg160
			sE-prot2 Glu144	H-bond	sE-prot2 Arg2 (mc)
sE-prot2 Thr147 (mc)	No H-bond		sE-prot2 Thr147 (mc)	H-bond	sE-prot2 Asp149
			sE-prot2 Thr147 (mc)	H-bond	sE-prot2 His157
sE-prot2 Asp149	H-bond	sE-prot2 Gly369 (mc)	sE-prot2 Asp149	H-bond	sE-prot2 Gly369 (mc)
			sE-prot2 Asp149	H-bond	sE-prot2 Thr147 (mc)
sE-prot2 Tyr150	H-bond	sE-prot2 Glu155	sE-prot2 Tyr150	No H-bond	
sE-prot2 Glu155	salt-bridge	pr Arg67	sE-prot2 Glu155	H-bond	sE-prot1 His104
			sE-prot2 Glu155	H-bond	sE-prot2 Ala152 (mc)
sE-prot2 His157	salt-bridge	pr Glu58	sE-prot2 His157	H-bond	sE-prot2 Thr147
			sE-prot2 His157	H-bond	sE-prot2 Gly159 (mc)
sE-prot2 Arg160	salt-bridge	pr Glu58	sE-prot2 Arg160	salt-bridge	sE-prot2 Glu144
***fg*-loop**			***fg*-loop**		
sE-prot2 Glu 207	H-bond	sE-prot1 His62	sE-prot2 Glu 207	No H-bond	
sE-prot2 His208 (mc)	H-bond	sE-prot1 Asn256 (mc)	sE-prot2 His208 (mc)	H-bond	sE-prot1 Asn256 (mc)
sE-prot2 His208	H-bond	sE-prot1 Asp253			
**domain 3**			**domain 3**		
sE-prot2 Lys315	H-bond	sE-prot2 Thr331	sE-prot2 Lys315	H-bond	sE-prot2 Glu329
sE-prot2 Lys315	H-bond	pr Gln 55 (mc)			
sE-prot2 Arg316	salt-bridge	sE-prot2 Glu329	sE-prot2 Arg316	salt-bridge	sE-prot2 Glu329
sE-prot2 Arg316	salt-bridge	pr Glu57			
sE-prot2 Glu 329	H-bond	sE-prot2 Lys 315	sE-prot2 Glu 329	salt-bridge	sE-prot2 Arg316
sE-prot2 Glu 329	H-bond	pr Glu57			

mc : atom from main chain
