# Supplementary material for: Evolution and activation mechanism of the flavivirus class II membrane-fusion machinery
Source: Nat Commun. 2022 Jun 28;13:3718. doi: 10.1038/s41467-022-31111-y (PMC9239988; doi:10.1038/s41467-022-31111-y)
Supplement: Supplementary file 3 — Reporting Summary [file 41467_2022_31111_MOESM3_ESM.pdf]

nature portfolio

Corresponding author(s): K. Stiasny, F.X. Heinz, F.A. Rey

Last updated by author(s): 2022/05/14

Reporting Summary

Nature Portfolio wishes to improve the reproducibility of the work that we publish. This form provides structure for consistency and transparency in reporting. For further information on Nature Portfolio policies, see our Editorial Policies and the Editorial Policy Checklist.

Statistics

For all statistical analyses, confirm that the following items are present in the figure legend, table legend, main text, or Methods section.

n/a	Confirmed	
☐	☒	The exact sample size (*n*) for each experimental group/condition, given as a discrete number and unit of measurement
☐	☒	A statement on whether measurements were taken from distinct samples or whether the same sample was measured repeatedly
☒	☐	The statistical test(s) used AND whether they are one- or two-sided<br>*Only common tests should be described solely by name; describe more complex techniques in the Methods section.*
☒	☐	A description of all covariates tested
☒	☐	A description of any assumptions or corrections, such as tests of normality and adjustment for multiple comparisons
☐	☒	A full description of the statistical parameters including central tendency (e.g. means) or other basic estimates (e.g. regression coefficient) AND variation (e.g. standard deviation) or associated estimates of uncertainty (e.g. confidence intervals)
☒	☐	For null hypothesis testing, the test statistic (e.g. *F*, *t*, *r*) with confidence intervals, effect sizes, degrees of freedom and *P* value noted<br>*Give P values as exact values whenever suitable.*
☒	☐	For Bayesian analysis, information on the choice of priors and Markov chain Monte Carlo settings
☒	☐	For hierarchical and complex designs, identification of the appropriate level for tests and full reporting of outcomes
☒	☐	Estimates of effect sizes (e.g. Cohen's *d*, Pearson's *r*), indicating how they were calculated

Our web collection on statistics for biologists contains articles on many of the points above.

Software and code

Policy information about availability of computer code

Data collection	Diffraction data for the 3 structures were collected using MXCuBE program at synchrotron beamlines PX1 (SOLEIL, Saint Aubin, France) and ID29 and ID23-1 (ESRF, Grenoble, France).
Data analysis	1. MALS data were analysed with ASTRA software (Wyatt Technolohy).<br>2. SPR data were processed using the Biacore T200 evaluation software (GE Healthcare), assuming a 1:1 interaction between E and pr.<br>3. X-ray data were indexed, integrated, scaled using the programs XDS (xds.mr.mpg.de/).<br>4. X-ray data were merged using the programs AIMLESS (CCP4 suite of programs).<br>5. X-ray data for 7QRF were corrected from the anisotropy using the programs DEBYE and STARANISO (Global Phasing Ltd, staraniso.globalphasing.org).<br>6. Molecular replacements were done with PHASER (from CCP4 suite of programs).<br>7. Structures were refined with BUSTER/TNT (Global Phasing Ltd, www.globalphasing.com/buster/)<br>8. All models were corrected with COOT (www2.mrc-lmb.cam.ac.uk/personal/pemsley/coot/).<br>9. Final models were analyzed with MolProbity (molprobity.biochem.duke.edu/)<br>10. All structure figures were created using PyMOL (Schrödinger, LLC, pymol.org/2/).<br>11. Electrostatic surfaces were computed with APBS, PDB2PQR and PROPKA software (server.poissonboltzmann.org/).<br>12. Polar contacts were computed with 'Protein interfaces, surfaces and assemblies' service PISA (www.ebi.ac.uk/pdbe/prot_int/pistart.html).<br>13. Multiple sequence alignments were calculated using ClustalW and displayed with ESPript (espript.ibcp.fr).<br>14. Variability of the flavivirus sequences was computed with ConSurf server (consurf.tau.ac.il/).

For manuscripts utilizing custom algorithms or software that are central to the research but not yet described in published literature, software must be made available to editors and reviewers. We strongly encourage code deposition in a community repository (e.g. GitHub). See the Nature Portfolio guidelines for submitting code & software for further information.

Data

Policy information about availability of data

All manuscripts must include a data availability statement. This statement should provide the following information, where applicable:

- Accession codes, unique identifiers, or web links for publicly available datasets
- A description of any restrictions on data availability
- For clinical datasets or third party data, please ensure that the statement adheres to our policy

Protein Data Bank accession codes: 7QRE (DOI:10.2210/pdb7qre/pdb), 7QRF (DOI:10.2210/pdb7qrf/pdb), 7QRG (DOI:10.2210/pdb7qrg/pdb). https://www.rcsb.org/.
GenBank accession numbers U27495, DQ235151, DQ235153, NC_001809,DQ235152, AY4386261, AF253419, AF331718, JF416959, L06436, AF311056, DQ235149, DQ235145, X03700, AF206518, AF315119, D00246, AF161266, AY453412, DQ359217, KJ776791, AB189120, M19197, AF349753, AY618991.

Field-specific reporting

Please select the one below that is the best fit for your research. If you are not sure, read the appropriate sections before making your selection.

☒ Life sciences ☐ Behavioural & social sciences ☐ Ecological, evolutionary & environmental sciences

For a reference copy of the document with all sections, see nature.com/documents/nr-reporting-summary-flat.pdf

Life sciences study design

All studies must disclose on these points even when the disclosure is negative.

Sample size	For SPR, a sample size of 2 was chosen as the independent measurements reported consistency and an accuracy in accordance with a standard pipetting error below ±10%.
Data exclusions	Data were not excluded from analysis.
Replication	SPR binding assays were performed twice, using independent samples. All attempts at replication were successful.<br>MALS: all attempts at replication were successful, and/or coincided with previously published data and/or the expected molecular weight as derived from the amino-acid composition.
Randomization	Randomisation is not relevant to our studies because of their biophysics nature.
Blinding	Blinding was not relevant to our studies because of their biophysics nature.

Reporting for specific materials, systems and methods

We require information from authors about some types of materials, experimental systems and methods used in many studies. Here, indicate whether each material, system or method listed is relevant to your study. If you are not sure if a list item applies to your research, read the appropriate section before selecting a response.

Materials & experimental systems

n/a	Involved in the study	
☒	☐	Antibodies
☐	☒	Eukaryotic cell lines
☒	☐	Palaeontology and archaeology
☒	☐	Animals and other organisms
☒	☐	Human research participants
☒	☐	Clinical data
☒	☐	Dual use research of concern

Methods

n/a	Involved in the study	
☒	☐	ChIP-seq
☒	☐	Flow cytometry
☒	☐	MRI-based neuroimaging

Eukaryotic cell lines

Policy information about cell lines

Cell line source(s)	Drosophila Schneider 2 cell line used as a recombinant production factory.
Authentication	The cell lines was not authenticated.
Mycoplasma contamination	Cell lines were not tested for mycoplasma contamination.
Commonly misidentified lines (See ICLAC register)	No commonly misidentified cell lines were used.
