# Supplementary figures and images for: Evolution and activation mechanism of the flavivirus class II membrane-fusion machinery
Source: Nat Commun. 2022 Jun 28;13:3718. doi: 10.1038/s41467-022-31111-y (PMC9239988; doi:10.1038/s41467-022-31111-y)

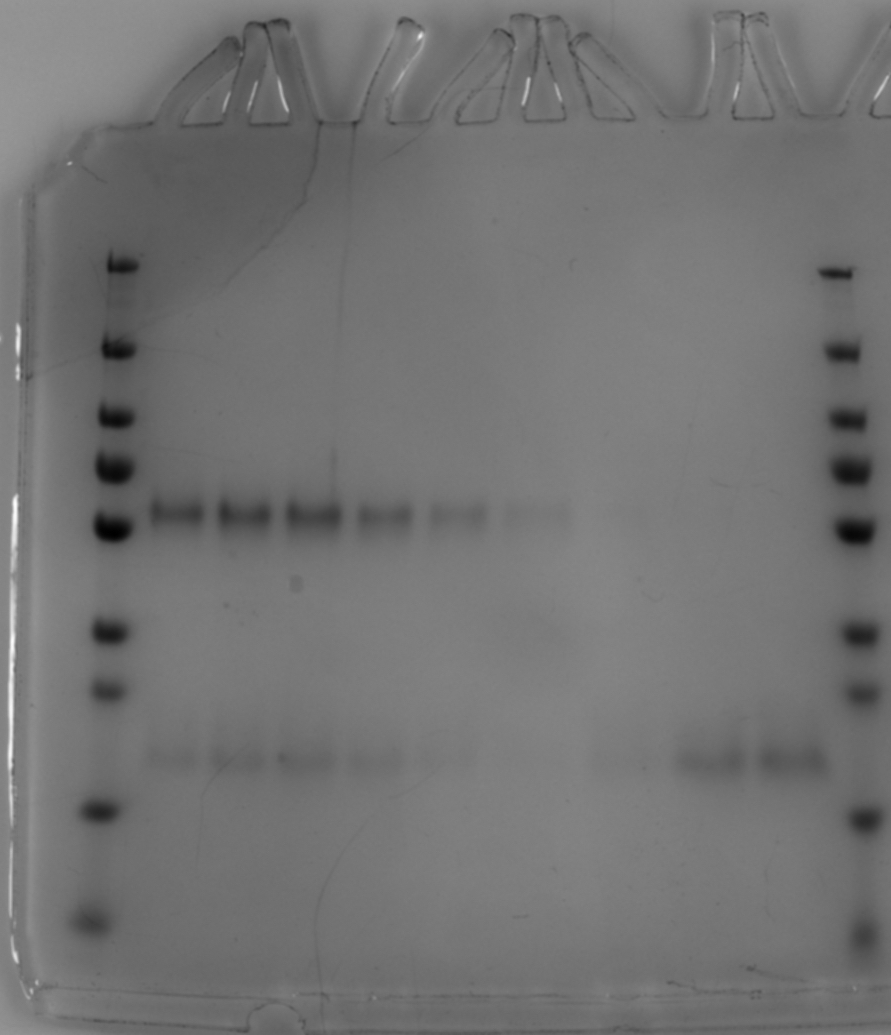

Supplement: Supplementary file 4 — Source Data [file 41467_2022_31111_MOESM4_ESM.zip › Source Data Fig 1c SDS PAGE pH 5.5.jpg]

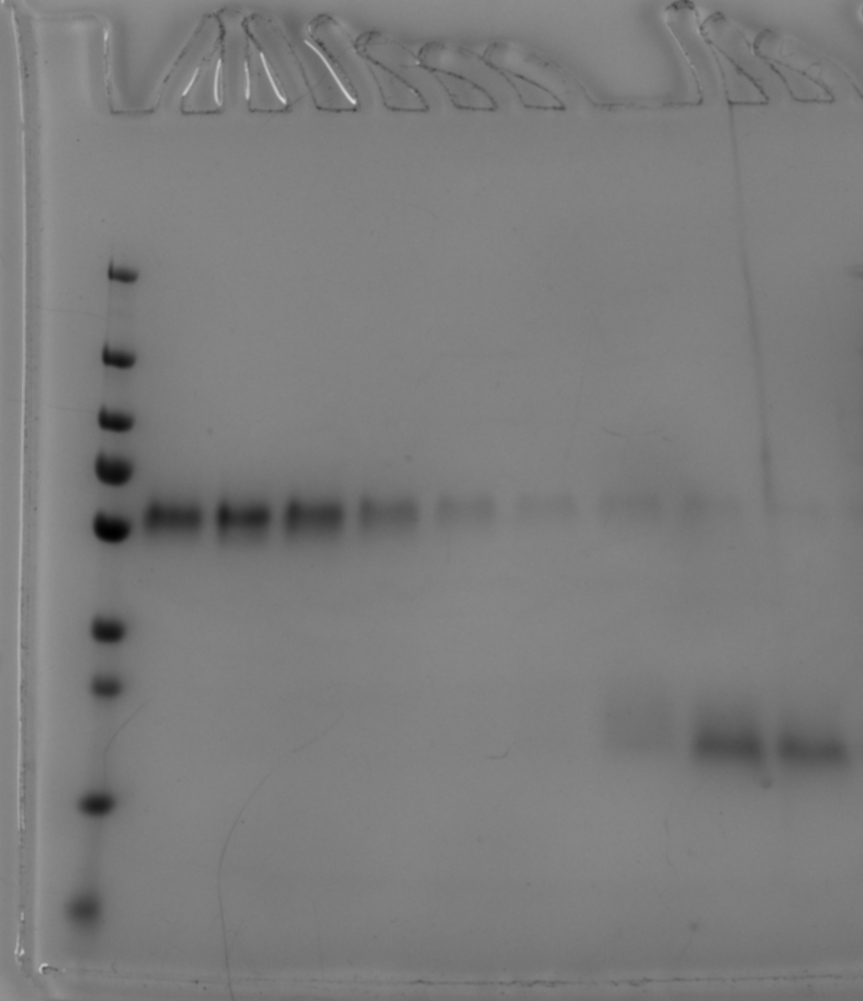

Supplement: Supplementary file 4 — Source Data [file 41467_2022_31111_MOESM4_ESM.zip › Source Data Fig 1c SDS PAGE pH 8.jpg]
